# Extract nonlinear operating rules of multi-reservoir systems using an efficient optimization method

**DOI:** 10.1038/s41598-022-21635-0

**Published:** 2022-11-07

**Authors:** Iman Ahmadianfar, Arvin Samadi-Koucheksaraee, Masoud Asadzadeh

**Affiliations:** 1 Department of Civil Engineering, Behbahan Khatam Alanbia University of Technology, Behbahan, Iran; 2 Department of Civil Engineering, University of Manitoba, Winnipeg, MB Canada

**Keywords:** Environmental sciences, Environmental social sciences, Energy science and technology, Engineering, Mathematics and computing

## Abstract

Hydropower plants are known as major renewable energy sources, usually used to meet energy demand during peak periods. The performance of hydropower reservoir systems is mainly affected by their operating rules, thus, optimizing these rules results in higher and/or more reliable energy production. Due to the complex nonlinear, nonconvex, and multivariable characteristics of the hydropower system equations, deriving the operating rules of these systems remains a challenging issue in multi-reservoir systems optimization. This study develops a self-adaptive teaching learning-based algorithm with differential evolution (SATLDE) to derive reliable and precise operating rules for multi-reservoir hydropower systems. The main novelty of SATLDE is its enhanced teaching and learning mechanism with three significant improvements: (i) a ranking probability mechanism is introduced to select the learner or teacher stage adaptively; (ii) at the teacher stage, the teaching mechanism is redefined based on learners’ performance/level; and (iii) at the learner stage, an effective mutation operator with adaptive control parameters is proposed to boost exploration ability. The proposed SATLDE algorithm is applied to the ten-reservoir benchmark systems and a real-world hydropower system in Iran. The results illustrate that the SATLDE achieves superior precision and reliability to other methods. Moreover, results show that SATLDE can increase the total power generation by up to 23.70% compared to other advanced optimization methods. Therefore, this study develops an efficient tool to extract optimal operating rules for the mentioned systems.

## Introduction

Hydropower is one of the most common sources of renewable energy, which is highly flexible to generate and has low environmental contamination compared with the fossil^1–4^. Most hydroelectricity is generated at large dams in the world, and several of the largest hydropower dams are located in the southern Iran. A dam reservoir maintains water to different target elevations such as flood control, hydropower production, and supplying irrigation demands^2,5,6^. A multi-reservoir system is a set of reservoirs placed in the same river basin. Optimizing the systems in terms of operation is useful for the comprehensive development of the river basin^7,8^. Extracting optimal operating rules for multi-reservoir hydropower systems is considered an arduous engineering problem because of the following reasons: (1) its optimization problem is commonly large-scale with a lot of constraints and unknown variables^9^; (2) due to different operational and physical constraints and opposite objectives^10^, it is not simple to achieve a suitable solution that meets all constraints^11^ and (3) the hydropower systems are nonconvex, nonlinear and non-differentiable^12^.

To optimize the problems related to the hydropower reservoir operation systems different types of optimization methods have been developed. In general, the optimization methods can be classified into three categories: (1) classical (traditional) techniques, (2) metaheuristic algorithms, and (3) hybrid algorithms. Traditional methods include linear programming (LP), nonlinear programming (NLP), and dynamic programming (DP). A metaheuristic optimization algorithm is a contemporary approach for guiding the search toward the optimal sections of the decision space in a given situation. A lot of various metaheuristic algorithms have been developed, comprising genetic algorithm (GA)^13^, differential evolution (DE)^14^, particle swarm optimization (PSO)^15^, gravitational search algorithm (GSA)^16^, cuckoo search (CS)^17^, artificial bee colony (ABC)^18^, gradient-based optimizer (GBO)^19^, Runge–Kutta optimization (RUN)^20^, and weighted mean of vectors (INFO)^21^.

Metaheuristic techniques are substantially flexible beyond the classical optimization ones because they do not need the constraints and fitness functions to be differentiable, convex, and linear, and therefore the methods can be taken on for solving a mixed variety of optimization problems. In this regard, these techniques are not able to ensure the global optimum, but they globally search for it and therefore are expected to find a near-optimum solution. Also, these approaches can readily be coupled with simulation models without the requirement for making any simplifying assumption^22–26^. Hybrid optimization algorithms use the strengths of various algorithms to achieve a more powerful optimization algorithm. For instance, Ahmadianfar et al. developed a hybrid optimization algorithm called A-DEPSO (i.e., Adaptive Differential Evolution with Particle Swarm Optimization) by combining the DE algorithm with PSO to optimize nonlinear and nonconvex hydropower systems^27^.

By 2021, a wide range of optimization procedures have been proposed to solve hydropower systems. Zhang et al. developed a multi-elite guide PSO (MGPSO) approach to maximize the energy provided by a multi-reservoir system consisting of ten cascaded hydroelectric units^28^. Asadzadeh et al. applied the Pareto Archived Dynamically Dimensioned Search heuristic multi-objective optimization algorithm to design the operation of the Great Lakes of the North America^8^. Taghian et al. used the GA algorithm to extract optimal hedging rule curves for a set of reservoirs built in a river in Iran^29^. Ahmadianfar et al. improved the bat algorithm (BA) utilizing the DE algorithm and used to find better solutions for sophisticated multi-reservoir systems^30^. Bozorg-Haddad et al. applied the biogeography-based optimization (BBO) to solve a single- and multi-reservoir system in Iran^31^. Their results confirmed the ability of the BBO algorithm to optimize the systems. Moravej and Hosseini-Moghari assessed the interior search algorithm (ISA) to solve problems in water resource management, and demonstrated that the ISA has an efficient performance to optimize complicated multi-reservoir problems^32^. Ehteram et al. optimized operation of the single- and multi-reservoir systems using shark algorithm (SA)^32^. They showed that the SA has the better efficiency to solve reservoir operation problems than the PSO and GA methods. Ahmadianfar et al. applied a powerful optimization technique namely hybrid of differential evolution (DE) and particle swarm optimization (PSO) with multi-strategy (MS-DEPSO) to solve multi-reservoir systems, which have purpose of hydropower generation and irrigation supply^23^. The outcomes indicated that the proposed optimizer can effectively extract operating rules for multi-reservoir systems. Liu et al. evaluated the ability of lion swarm optimization (LSO) algorithm to optimize the dispatch of cascade hydropower stations located in China^33^. They demonstrated that the LSO algorithm has a better efficiency than the GA, PSO, and improved cuckoo search (CS) algorithms in terms of reliability and accuracy. Feng et al. developed a modified version of sine cosine algorithm (SCA) called quasi-opposition SCA (QSCA) to precision optimization of multiple hydropower reservoir systems^4^. Their findings demonstrated that the suggested QSCA approach, which is based on the convergence rate and the quality of the solution, is a dependable and resilient method. It should be noted that all the studies mentioned above were single-objective. In applying optimization methods in the multi-objective water resources problems, many studies, such as Ahmadianfar et al., introduced a novel multi-objective PSO with DE algorithm (MOPSO-DE) to optimize a multi-reservoir and multi-objective system in Iran^34^. They showed that the proposed method could significantly mitigate severe drought periods. Hatamkhani and Moridi applied the MOPSO algorithm and linked it to the water evaluation and planning system (WEAP) model to optimize long-term planning at a basin in Iran^35^. The findings proved that the simulation–optimization model performed its duties correctly in the context of the best distribution and planning of water resources within the basin. Fang and Popole developed an efficient multi-objective PSO (MOPSO) improved by adopting self-organizing mapping (SOM) method^36^. They applied the proposed algorithm on a hydropower system in China. The findings demonstrate that the model can obtain the best schedule by considering the environmental advantages and the benefits of power production. Despite the considerable success that has been obtained in practice, the utilization of the above optimizers is still confined by some drawbacks, such as parameter tuning, dimensionality course, and premature convergence^4,21,23,37–40^.

Teaching–learning-based optimization (TLBO) introduced by Ref.^41^ emulates the teaching–learning procedure in a class. TLBO is an effective and simple optimizer with only one parameter tuning (i.e., the size of population). Several variants of TLBO have been recently developed, e.g. generalized oppositional TLBO (GOTLBO)^42^, self-adaptive TLBO (SATLBO)^43^, and improved TLBO (ITLBO)^44^. However, these variants of the TLBO algorithm suffer from low reliability, insufficient precision, especially for complex and high dimensional problems^38^.

SATLDE is developed in this study to derive reliable and precise operating rules of hydropower reservoir operation systems. SATLDE uses a ranking mechanism to adaptively select a learner or teacher stage, instead of selecting both stages, to reduce the computing cost significantly, in each iteration. At the teacher stage, the teaching mechanism is enhanced to teach learners based on the level (performance) of each learner, aiming to assist all learners to achieve promising levels. Also, at the learner stage, an effective mutation operator is introduced based on the DE algorithm with self-adaptive control parameters to promote population diversity and boost wider global search ability, avoiding TLBO from being trapped into local optimal solutions. This paper evaluates the performance of SATLDE in the context of addressing benchmark and real-world multi-reservoir hydropower system problems.

The structure of paper can be arranged as follows. “Methodology” section expresses the TLBO and SATLDE algorithms. The performance criteria of optimization method are described in “Teaching learning-based optimization” section. The mathematical formulation and outcomes of ten-reservoir ordeals is illustrated in “Performance assessment of the optimizers” section. “Results and discussion” section formulates a real-world case study with four reservoirs, stating the experimental outcomes. In the last Section, results and complementary information are given.

## Methodology

### Multi-reservoir system modeling

Defining the operating rules of hydropower reservoir systems has the most significant impact on the performance of these systems; improving these rules could lead to an increased energy output that is either more dependable or more consistent. Specifying operating rules for these systems continues to be a challenging topic in multi-reservoir system optimization because of the complicated nonlinear and multivariable properties of the equations that make up the hydropower system. Therefore, providing an efficient optimization algorithm to solve these systems is an inevitable task. In this section, a benchmark multi-reservoir system with ten reservoir is modeled and the performance of proposed optimization method is assessed on this problem. Finally, optimal operating rules are extracted using the proposed optimization method for a real-world multi-reservoir system in Iran.

### Ten-reservoir system

**Figure 1:**
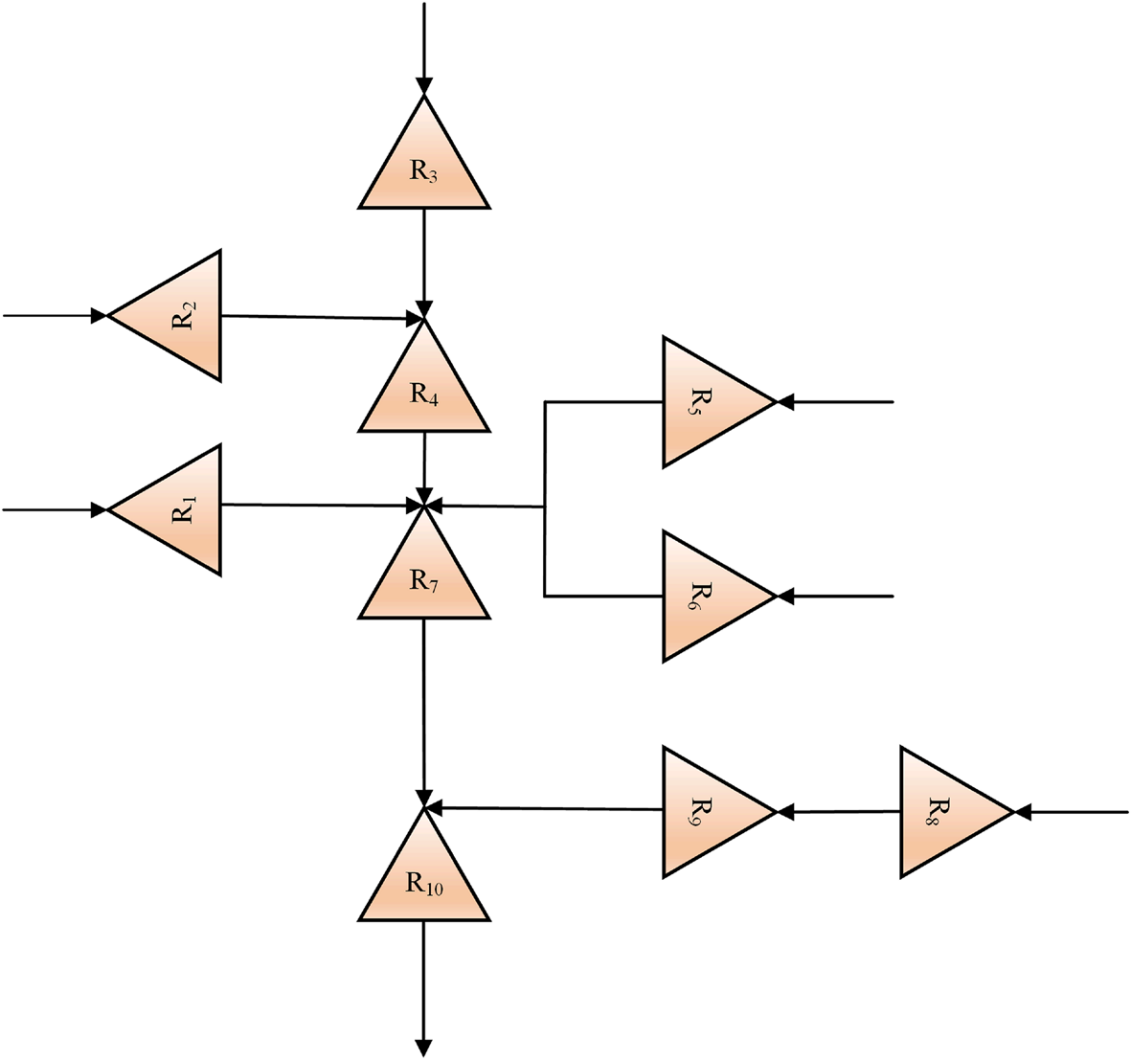
Schematic of ten-reservoir problem.

A well-known hypothetical multi-reservoir system namely the ten-reservoir system introduced by Ref.^45^ is solved by all optimization algorithms introduced in the previous section. The problem is substantially more complex that the four-reservoir problem due to the large number of constraints and decision variables. Figure 1 displays the schematic of this system. As it can be seen from this figure, the system has 10 reservoirs with the purpose of hydropower generation at 12-time steps. Detail of this system were provided in Ref.^45^. The fitness function of the problem is defined by Eq. (1),

1 $$Maximize\, F= \sum_{l=1}^{L}\sum_{t=1}^{T}[{C}_{t}^{l}\times {R}_{t}^{l}],$$

where $F$ denotes the fitness function, $l$ denotes reservoir number, $L$ is known as the total number of reservoirs, $T$ denotes the total number of simulation periods, ${C}_{t}^{l}$ is stated as the benefit of *l*th reservoir at period *t*, ${R}_{t}^{l}$ is the *l*th reservoir release amount at period *t*.

The main constraints of this system are described by Eqs. (2)–(5),

2 $${V}_{t+1}^{l}={V}_{t}^{l}+{In}_{t}^{l}-{R}_{t}^{l}\quad\text{ for }l=1,\dots , L, t=1,\dots , T,$$

3 $${R}_{min}^{l}\le {R}_{t}^{l}\le {R}_{max}^{l}\quad\text{ for }l=1,\dots , L, t=1,\dots , T,$$

4 $${V}_{min}^{l}\le {V}_{t}^{l}\le {V}_{max}^{l}\quad\text{ for }l=1,\dots , L, t=1,\dots , T$$

5 $${V}_{T+1}^{l}={V}_{1}^{l}\quad\text{ for }l=1,\dots , L,$$

where ${V}_{t+1}^{l}$ and ${V}_{t}^{l}$ are the volume of *l*th reservoir storage at period *t* + 1 and *t* respectively, ${In}_{t}^{l}$ denotes the amount of inflow into *l*th storage at period *t*, ${R}_{min}^{l}$ and ${R}_{max}^{l}$ denote the minimum and maximum volume of release form *l*th reservoir storage at period *t*, ${V}_{min}^{l}$ and ${V}_{max}^{l}$ denote the lowest and highest volume of storage of *l*th reservoir storage at period span *t*. In this problem, the main decision variables are considered the amount of reservoir release (${R}_{t}^{l}$) and the state variables are the volume of reservoir storage (${V}_{t}^{l}$). Therefore, the constraints related to releases are directly handled by the proposed optimizer, while the constraints applied to the reservoir storage volumes are embedded as the penalty functions (PFs). In this regard, the fitness function defined in Eq. (1) can be redefined by embedding the following PFs,

6 $$PF_{1} = \left\{ {\begin{array}{*{20}l} {d_{1} \times (V_{{T + 1}}^{l} - V_{1}^{l} )^{2} } \hfill & {if\,V_{{T + 1}}^{l} \ne V_{1}^{l} } \hfill \\ 0 \hfill & {otherwise} \hfill \\ \end{array} ,} \right.$$

7 $$PF_{2} = \left\{ {\begin{array}{*{20}l} {d_{2} \times (V_{{min}}^{l} - V_{t}^{l} )^{2} } \hfill & {if\, V_{t}^{l} < V_{{min}}^{l} } \hfill \\ {d_{3} \times (V_{{min}}^{l} - V_{t}^{l} )^{2} } \hfill & {if\, V_{t}^{l} > V_{{max}}^{l} } \hfill \\ 0 \hfill & {otherwise} \hfill \\ \end{array} } \right.,$$

where ${d}_{1}$, ${d}_{2}$, and ${d}_{3}$ are the PF coefficients and are equal to 60^22,30^. Accordingly, the fitness function can be expressed as,

8 $$Maximize\, F= \sum_{l=1}^{L}\sum_{t=1}^{T}[{C}_{t}^{l}\times {R}_{t}^{l}]-\left(PF1+PF2\right).$$

### Real-world multi-reservoir system

**Figure 2:**
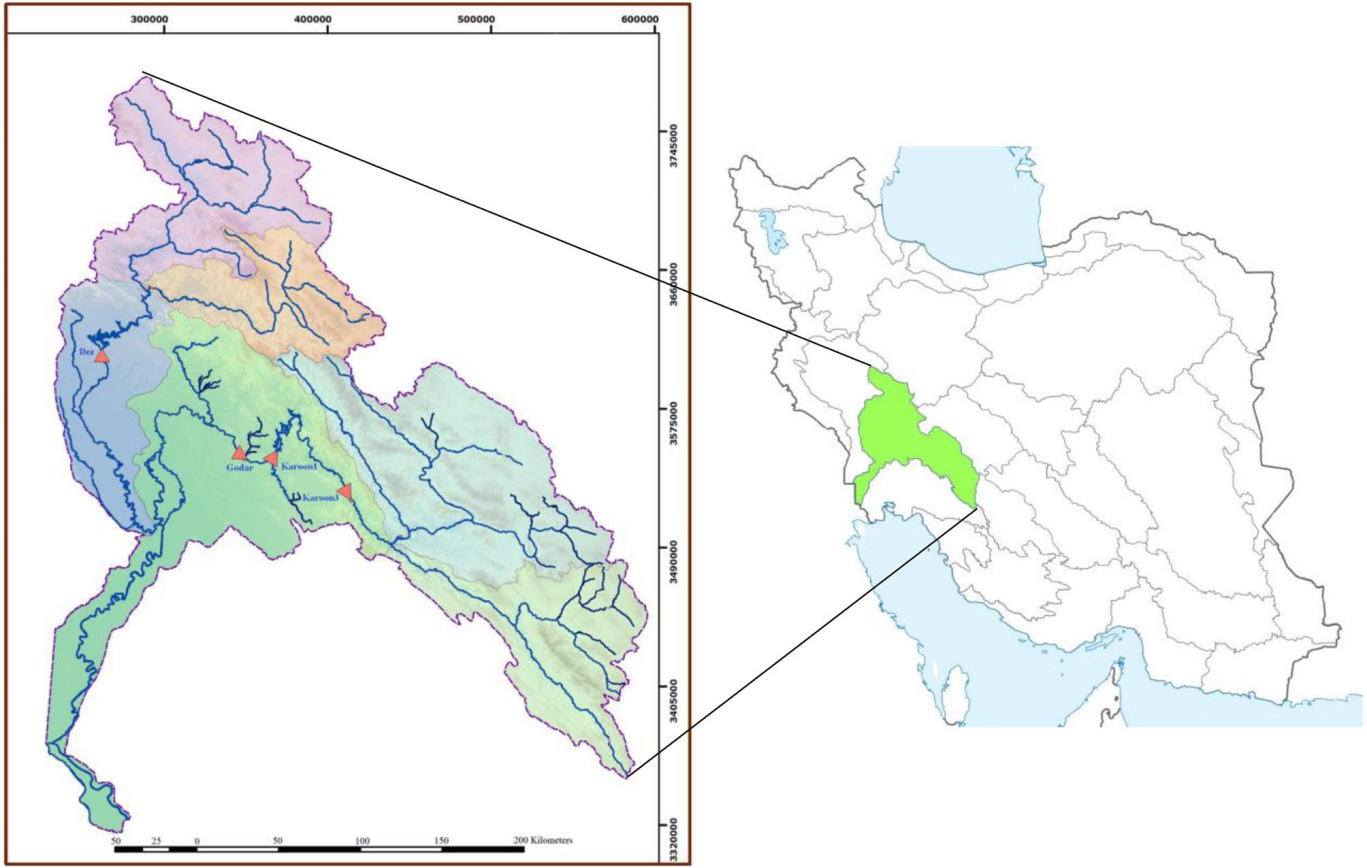
Location of Karoon–Dez basin.

**Figure 3:**
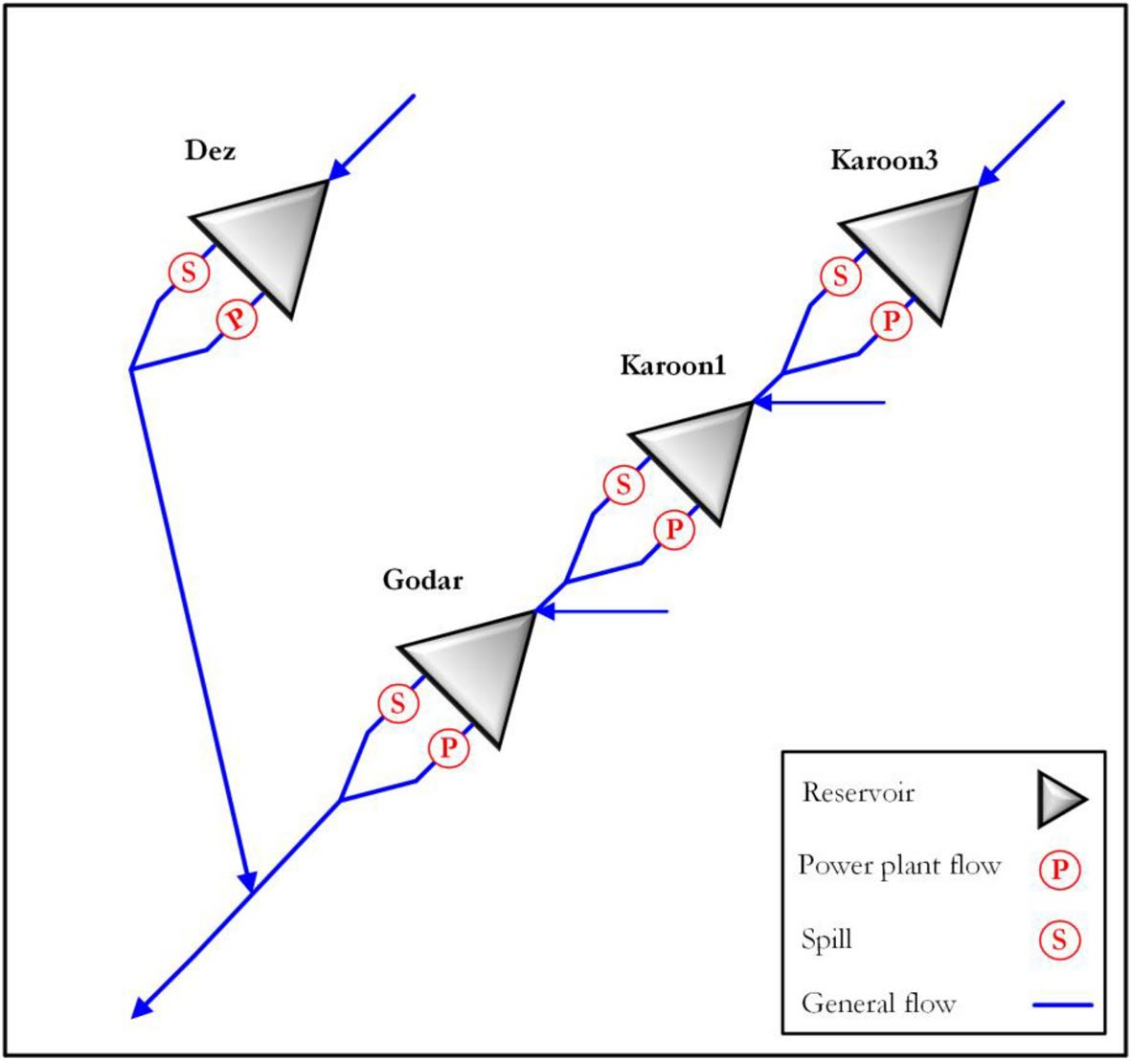
Diagram of the real-world multi-reservoir system.

**Figure 4:**
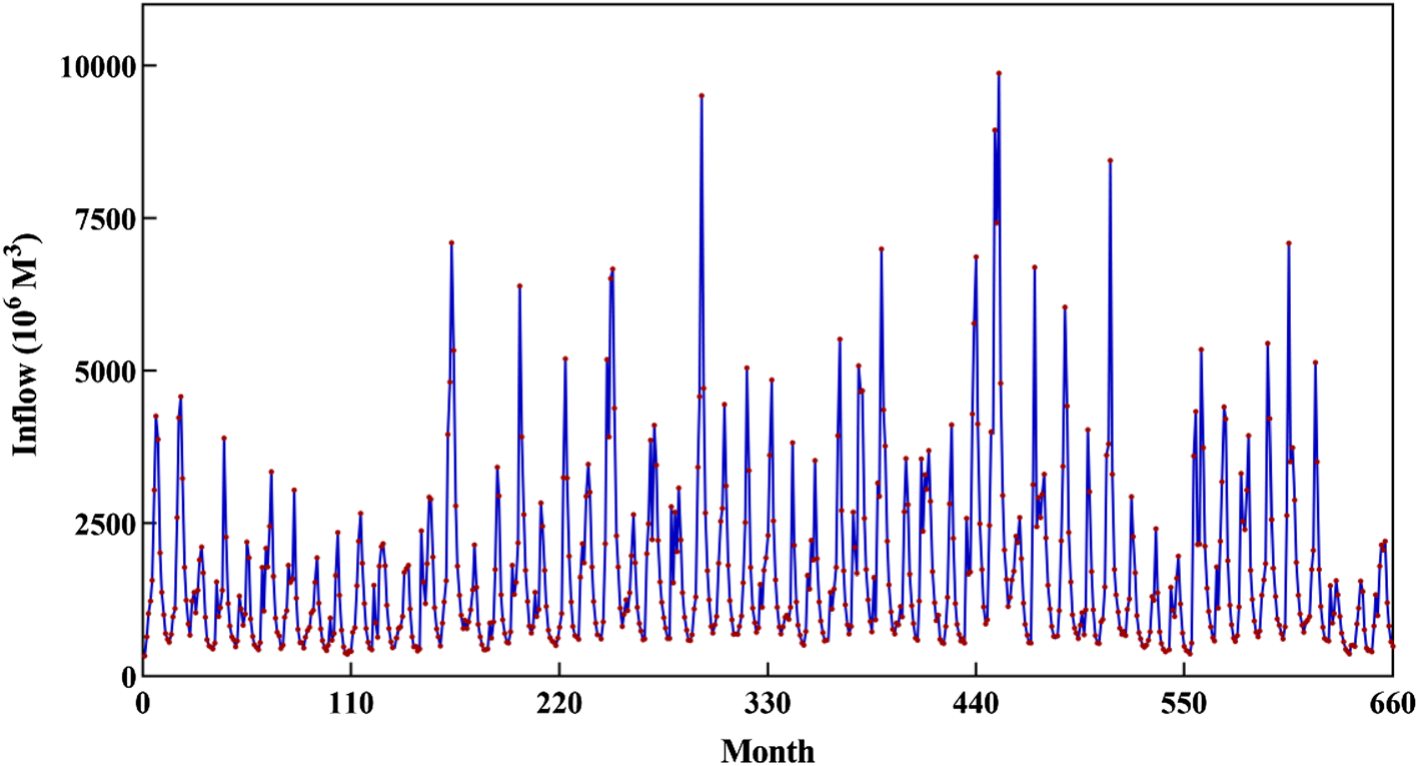
Monthly inflow into the multi-reservoir system.

In the current section, a real-world multi-reservoir system with the hydropower generation purpose is considered for examining the performance of SATLDE. This multi-reservoir system is situated in the Karoon–Dez (KD) basin, the southwest of Iran. In the KD basin, there are four reservoirs including Karoon3, Karoon1, Godar, and Dez. The map of study area is depicted in Fig. 2. This system was optimized during a 55-year operational time period (monthly time step). The diagram of this four-reservoir system is displayed in Fig. 3. In addition, the monthly inflow into the system is depicted in Fig. 4.

#### Mathematical model of a multi-reservoir system

The mathematical formulation of the multi-reservoir system operation is explained in this section. It should be noted that the proposed problem was simplified and turned into a single objective problem. Therefore, the fitness function and constraints are formulated based on this simplification. It is worth noting that the main goal of the proposed four-reservoir system is power production, so we formulated the problem based on maximizing power generated at each time step (monthly). The fitness (objective) function, limitations, and the proposed operating rule are all specified in great details.

#### Fitness function

The fitness function of the multi-reservoir hydropower system utilized here is to maximize the total power generated of the mentioned system, subject to different physical and technical constraints. In this research, the fitness function is to reach the lowest disparity between the maximum power produced by power plants and power generated by each of them over the operation periods, which is defined below,

9 $$Minimize \,Z=\sum_{l=1}^{L}\sum_{t=1}^{T}{\left[1-\frac{{Power}_{t}^{l}}{{Power}_{max}^{l}}\right]}^{2},$$

9-1 $${Power}_{t}^{k}=g\times {e}^{l}\times {R}_{t}^{l}\times {havg}_{t}^{l}\times \frac{1}{{f}^{l}\times 1000},$$

9-2 $${havg}_{t}^{k}=0.5\times {(h}_{t}^{l}+{h}_{t+1}^{l})-{htail}^{l},$$

where $Z$ denotes the fitness function, ${Power}_{t}^{l}$ is the energy yield of the *l*th reservoir over the *t*th time period, ${Power}_{max}^{l}$ denotes the maximum power produced by the *l*th power plant over the *t*th time period. $g$ is deemed 9.81, ${e}^{l}$ is the efficiency of *l*th power plant at the *t*th period, ${havg}_{t}^{l}$ denotes the water head of the *l*th power plant at the *t*th period, ${f}^{l}$ denotes the plant factor of the *l*th power plant, ${h}_{t}^{l}$ denotes the water level of *l*th reservoir during the *t*th time period, ${htail}^{l}$ is the downstream water level of *l*th reservoir.

#### Constraints

In this subsection, the main constraints of this problem are expressed as,

10 $${V}_{t+1}^{l}={V}_{t}^{l}+{\text{In}}_{t}^{l}-{R}_{t}^{l}-{Sp}_{t}^{l}-{Evp}_{t}^{l},$$

11 $$Sp_{t}^{k} = \left\{ {\begin{array}{*{20}l} {V_{{t + 1}}^{l} - V_{{max}}^{l} } & {if\, V_{{t + 1}}^{l} > V_{{max}}^{l} } \\ 0 & {otherwise} \\ \end{array} ,} \right.$$

12 $${Evp}_{t}^{l}={Area}_{t}^{l}\times {hevp}_{t}^{l},$$

13 $${{V}_{min}^{l}\le V}_{t}^{l}\le {V}_{max}^{l},$$

14 $${{R}_{min}^{l}\le R}_{t}^{l}\le {R}_{max}^{l},$$

15 $${V}_{T+1}^{l}={V}_{1}^{l},$$

where ${Sp}_{t}^{l}$ denotes the spill form the *l*th reservoir at the *t*th time period, ${Evp}_{t}^{l}$ denotes the evaporation volumes from the *l*th reservoir at the *t*th time period, ${Area}_{t}^{l}$ denotes the *l*th reservoir at the *t*th time period, ${hevp}_{t}^{l}$ denotes the evaporation level of the *l*th reservoir at the *t*th time period.

#### Nonlinear operating rule

For the multi-reservoir system, this section introduces a nonlinear operating rule. The rule is implemented by conditioning reservoir release on reservoir storage and inflow into each reservoir at each time step^46^. Based on this rule, a suitable regression analysis is used to fit a nonlinear relationship to the data, which is formulated as,

16 $${R}_{t}^{l}={\alpha }^{l}\times \left[\frac{\sqrt{{\left({V}_{t}^{l}-{V}_{min}^{l}\right)}^{2}+{In}_{t}^{l}}}{{u}^{l}+\sqrt{{\left({V}_{t}^{l}-{V}_{min}^{l}\right)}^{2}+{In}_{t}^{l}}}\right],$$

where ${\alpha }^{l}$ and ${u}^{l}$ are the main parameters of this rule for the *l*th reservoir, which are determined by the optimizer.

#### Simulation–optimization process

The main steps for extracting the optimal operating rules for the hydropower multi-reservoir system are described as,

- Generate a set of random values (96 decision variables: 24 variables for each reservoir) using the optimization method within a feasible range (feasible ranges for $\alpha$ and $u$ are (0, 1] and [− 400, 400], respectively) and calculating the reservoir release for each reservoir using Eq. (16).
- Enter the releases as the input to the reservoir simulation model to calculate the volume of storage at period t + 1 utilizing Eq. (10).
- Evaluate the storage volumes to be in the range of the allowable range.
- Save the values of each created releases if they are in the range of search space.
- Repeat steps (b)–(d) for calculating the volumes of releases and computing the fitness function for all time steps using Eq. (9). Next, the stopping criteria in the optimization process is evaluated, if it is met, then the simulation–optimization process is finished, else go to Step (b).

## Teaching learning-based optimization

TLBO is known as an effective optimization algorithm^41^. This algorithm mimics the teaching process in a class and is deemed a population-based optimizer. The optimization process in TLBO includes two main stages: the teacher stage (TS) and learner stage (LS). In the first stage (i.e., TS), teacher tries share its data with the existing learners and in the LS, learners teach each other^41^.

### Teacher stage

There is a teacher and *N*p − 1 learners in the TLBO algorithm, where *N*p is the number of members in a population. In TS, the teacher shares its information with learners to promote the mean score of the class. In this algorithm, the teaching action can be formulated as,

17 $${x}_{TS,k}={x}_{k}+{r}_{1}\times \left({x}_{T}-TF.{x}_{M}\right),$$

where ${x}_{TS,k}$ denotes the *k*th position calculated in TS, ${r}_{1}$ denotes a random number in the interval of [0, 1]. ${x}_{T}$ is the position of teacher (i.e., best-so-far position), $TF$ is the factor of teaching and is equal to $round(1+rand)$. $rand$ denotes a random number in the interval of [0, 1]. ${x}_{M}$ denotes the mean value of class, which is defined in Eq. (18).

18 $${x}_{M}=\frac{1}{Np}\sum_{k=1}^{K}{x}_{k}.$$

After the position of all learners was updated in TS, they are assessed by the fitness function $f(x)$. If $f({x}_{TS,k})$ is better than $f({x}_{k})$, then ${x}_{k}$ is substituted with ${x}_{TS,k}$; otherwise, ${x}_{k}$ is maintained.

### Learner stage

In LS, a learner boosts itself by choosing another random learner from the population, which is formulated in Eq. (19).

19 $$x_{{LS,k}} = \left\{ {\begin{array}{*{20}l} {x_{k} + r_{2} \times \left( {x_{k} - x_{l} } \right)} \hfill & {if{\mkern 1mu} f\left( {x_{k} } \right) < f(x_{l} )} \hfill \\ {x_{k} + r_{2} \times \left( {x_{l} - x_{k} } \right)} \hfill & {otherwise} \hfill \\ \end{array} ,} \right.$$

where ${x}_{l}$ is the *l*th learner and $l\ne k$. After creating the ${x}_{LS,k}$, if $f\left({x}_{LS,k}\right)$ is superior than $f\left({x}_{k}\right)$, then accept ${x}_{LS,k}$.

### Proposed self-adaptive teaching learning-based with differential evolution

The TLBO method is a simple and effective optimization algorithm that solves the problem by mimicking the teaching–learning process in a classroom. In the TLBO, the optimization process is implemented by the teacher and learner stages, bring along a lot of computational time (2*N*p) compared with other optimization algorithms^43^. In the TS, whereas the learner’s score is likely to be promoted, the enhancement of each learner’s score depends to some extent on their capability. In addition, in LS, a learner (${x}_{l}$) is randomly selected to share information, which may lead to confined learning capability and ultimately a weak global searchability (exploration)^42–44^. Accordingly, implementing a strategy that adaptively selects LT or LS based on the performance of learners can significantly decrease the computational time. Hence, TLBO is enhanced to the self-adaptive teaching learning-based with differential evolution (SATLDE) in this study, which is described in the subsequent sections.

#### Ranking probability strategy

Ranking probability strategy (RPS) proposed by Ref.^43^ is used in SATLDE in order to better identify the degrees of various learners. According to RPS, the whole of the learners is sorted from the best to the worst based on their objective function value as in Eq. (20),

20 $$Ind=sort\left(f, ascend\right),$$

where $Ind$ denotes the index of learners after sorting. $f$ denotes the objective function values.

Next, the ranking of all learners is calculated as in Eq. (21).

21 $$Rank\left(Ind\left(k\right)\right)=Np-k, k=1, 2, \dots , Np.$$

Finally, the ranking probability of each learner ($Rankp$) is formulated as Eq. (22).

22 $$Rankp\left(Ind\left(k\right)\right)={\left(\frac{Rank\left(Ind\left(k\right)\right)}{Np}\right)}^{2}, k=1, 2, \dots , Np.$$

According to Eq. (22), the better learners have the larger $Rankp$ while the worst learners have lower $Rankp$.

#### Teacher stage of SATLDE

The original TLBO uses the teacher (${x}_{T}$) and the mean level of learners (${x}_{M}$) for guiding all learners in the teacher stage (see Eq. (1)). Generally, learning levels of various learners is different from each other. Therefore, this study suggests employing an effective technique to assist diverse learners in achieving a better score. Accordingly, learners in a class can be divided into two groups. To specify these groups, ${x}_{M}$ has a key role. With this consideration, learners are located with the better learning level in the first group with condition $f\left({x}_{k}\right)<f({x}_{M})$, while the second group ($f\left({x}_{k}\right)\ge f({x}_{M})$) belongs to the worse learners. In the proposed strategy, better ones are guided by ${x}_{T}$, the teacher, and ${x}_{k}$, the current learner. Additionally, in order to stay away from local solutions, two learners are picked at random and utilized to conduct the better learners. The proposed teacher stage procedure in SATLDE is presented by script (S1). 
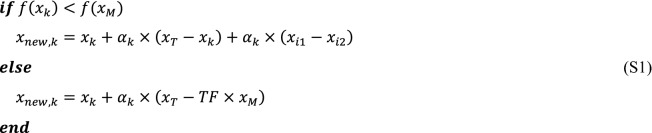
where $i1$ and $i2$ are two random integer number in the interval of [1, *N*p] and $i1\ne i2\ne k$. It is noteworthy that $i2$ is randomly selected from the union $P\cup Ar$, where $P$ is the current population and $Ar$ is a set of inferior solutions at each iteration^47^. ${\alpha }_{k}$ denotes the scale factor, which is calculated as follows,

23 $${\alpha }_{k}={\upsilon }_{\alpha }+0.1\times randn,$$

where $randn$ denotes a random number with a normal distribution. ${\upsilon }_{\alpha }$ is equal to 0.5 in the first iteration and updated by utilizing Eq. (24),

24 $${\upsilon }_{\alpha }=\left(1-b\right)\times {\alpha }_{k}+b\times {newS}_{\alpha },$$

24-1 $${newS}_{\alpha }=\frac{\sum_{m=1}^{M}{S}_{\alpha }^{2}}{\sum_{m=1}^{M}{S}_{\alpha }} ,$$

24-2 $$M=size\left({S}_{\alpha }\right),$$

24-3 $$b=\frac{median(df)}{\sum_{m=1}^{M}df},$$

where ${S}_{\alpha }$ is the set of all successful learner factors ${\alpha }_{k}$ at each iteration. $M$ is the size of ${S}_{\alpha }$. $df$ is the difference between the *k*th learner and successful learner and it can be defined according to the following script (S2).


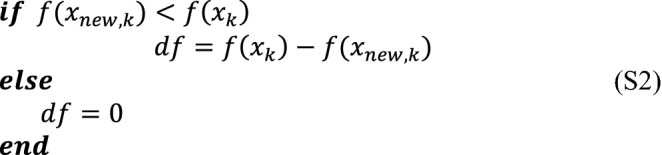


#### Learner stage of SATLDE

As stated in “Real-world multi-reservoir system” section, TLBO uses a random learner to find a better region in the search space, leading to confined learning capability and weak global search. Hence, it is critical for the TLBO to improve the efficiency of the learning stage. In this research, an efficient mutant vector is utilized to create the new learner ${x}_{new,k}$, which is expressed as,

25 $${x}_{new,k}={x}_{k}+{\alpha }_{k}\times \left({x}_{T}-{x}_{k}\right)+{\alpha }_{k}\times \left({x}_{i1}-{x}_{i2}\right).$$

According to Eq. (25), the knowledge of learners (${x}_{i1}$, ${x}_{i2}$, and ${x}_{k}$) can be fully used and an exploration capability can be guaranteed. The proposed algorithm uses random vectors (${x}_{i1}-{x}_{i2}$) to search globally in the solution space at each iteration. This mechanism is used to have a chance to explore most regions of the search space to find better solutions.

#### Crossover

In order to improve the population variety, crossover is one of the most often used operators^48^. This operator combines the new learner created by the TS or LS with the current learner at each iteration and generates a new learner, which is given as,

26 $${z}_{k,j}=\left\{\begin{array}{*{20}l}{x}_{new,k,j} & if\, rand<{Cr}_{k} or j=jr \\ {xp}_{k,j} & otherwise \end{array},\right.$$

in which

27 $${xp}_{k}=\left\{\begin{array}{*{20}l}{x}_{k} & if\, rand<\left(1-\frac{NFE}{MaxNFE}\right),\\ {x}_{lb} & otherwise\end{array}\right.$$

where $jr$ denotes an inter random number choosen from the interval of [1, D], $NFE$ is the number of function evaluations, $MaxNFE$ is maximum number of function evaluations, ${xp}_{k}$ is a learner which is specified from between ${x}_{k}$ and ${x}_{lb}$ based on the condition $rand<(1-\frac{NFE}{MaxNFE})$. Accordign to Eq. (14), in the first iteration the learner ${xp}_{k}$ is most probably equal to ${x}_{k}$ and in the last iteration it is most probably equal to ${x}_{lb}$. ${Cr}_{k}$ is the *k*th crossover rate, which is determined based on Eq. (28),

28 $${Cr}_{k}= {\upsilon }_{Cr}+0.1\times randn,$$

where ${\upsilon }_{Cr}$ is equal to 0.5 in the first iteration and updated by using Eqs. (29) and (29-1),

29 $${\upsilon }_{Cr}=\left(1-b\right)\times {\upsilon }_{Cr}+b\times new{S}_{Cr},$$

in which

**Figure 5:**
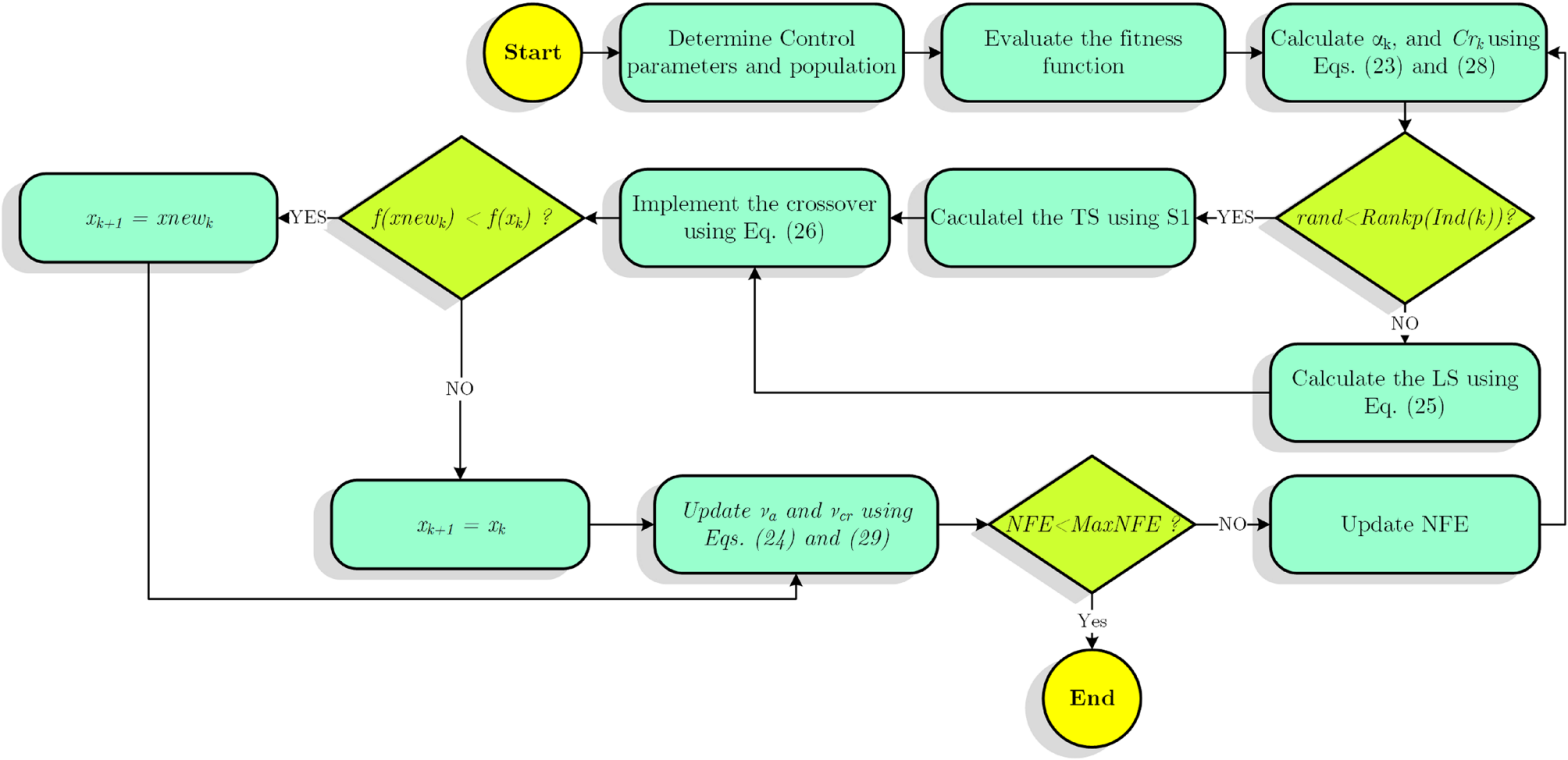
Flowchart of SATLDE.


29-1 $$new{S}_{Cr}=\frac{1}{M}\sum_{m=1}^{M}{S}_{Cr},$$

where ${S}_{Cr}$ denotes the set of all successful learner crossover rate (${Cr}_{k}$) at each iteration. Algorithm 1 gives the pseudo code of SATLDE algorithm. Also, the SATLDE flowchart is displayed in Fig. 5.

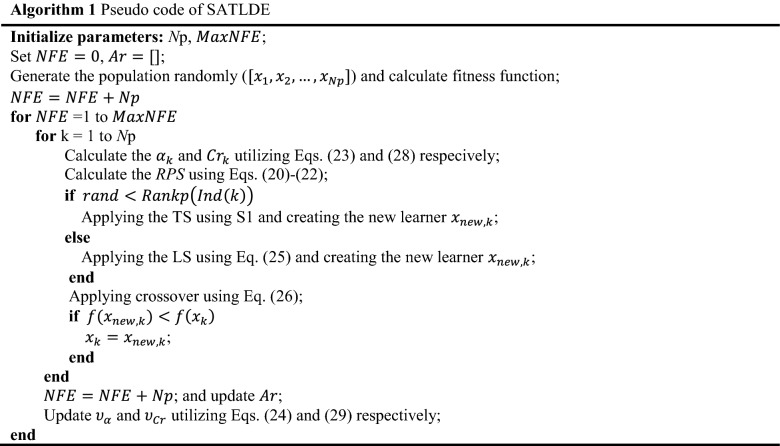


## Performance assessment of the optimizers

The correlation coefficient (R), root mean square error (RMSE), mean absolute error (MAE), mean absolute percentage error (MAPE), Willmott’s agreement Index (IA), the Legate and McCabe’s index ($\text{E}$), and centered root mean square error (cRMSE) are taken on to evaluate the efficiency of optimization methods, which are defined here.

30 $$R=\frac{\sum_{i=1}^{N}\left({R}_{LP,i}-\overline{{R }_{LP}}\right)\times ({R}_{OM,i}-\overline{{R }_{OM}})}{\sqrt{\sum_{i=1}^{N}{({R}_{LP,i}-\overline{{R }_{LP}})}^{2}\sum_{i=1}^{N}{({R}_{OM,i}-\overline{{R }_{OM}})}^{2}}},$$

31 $$RMSE={\left(\frac{1}{N}{\sum }_{i=1}^{N}{\left({R}_{LP,i}-{R}_{OM,i}\right)}^{2}\right)}^{0.5},$$

32 $$MAE=\left(\frac{1}{N}\right){\sum }_{i=1}^{N}\left|{R}_{LP,i}-{R}_{OM,i}\right|,$$

33 $$MAPE\left(\%\right)=\left(\frac{100}{N}\right){\sum }_{i=1}^{N}\left|\frac{{R}_{LP,i}-{R}_{OM,i}}{{R}_{LP,i}}\right|,$$

34 $$IA=1-\frac{\sum_{i}^{N}{\left({R}_{LP,i}-{R}_{OM,i}\right)}^{2}}{\sum_{i=1}^{N}{\left(\left|\left({R}_{LP,i}-\overline{{R }_{LP}}\right)\right|+\left|\left({R}_{OM,i}-\overline{{R }_{LP}}\right)\right|\right)}^{2}}, 0<{I}_{A}\le 1,$$

35 $$E=1-\frac{{\sum }_{i=1}^{N}\left|{R}_{LP,i}-{R}_{OM,i}\right|}{{\sum }_{i=1}^{N}\left|{R}_{LP,i}-\overline{{R }_{OM}}\right|},$$

where *N* is number of dataset (for four- and ten-reservoir problems is equal to 48 and 120, respectively), ${R}_{LP,i}$ denotes the reservoir release obtained by LP model, ${R}_{OM,i}$ denotes the reservoir release achieved by optimization methods, $\overline{{R }_{LP}}$ and $\overline{{R }_{OM}}$ denote the mean values of reservoir releases optimized by LP and optimization methods, respectively. Decisions based on these criteria are difficult, and for easier decision-making in order to select the best method, they can be turned into a single criterion. In this paper, a multi-index metric was applied to assess the performance of optimization methods, which is called the performance index (PI) and defined as,

36 $$PI=\frac{1}{6}\left(\frac{{R}_{min}}{R}+\frac{RMSE}{{RMSE}_{max}}+\frac{MAE}{{MAE}_{max}}+\frac{MAEP}{{MAEP}_{max}}+\frac{{E}_{min}}{E}+\frac{{IA}_{min}}{IA}\right),$$

where ${R}_{min}$, ${E}_{min}$, and ${IA}_{min}$ are the minimum value of $R$, $E$, and $IA$ calculated by all optimizers. Also, ${RMSE}_{max}$, ${MAE}_{max}$, and ${MAEP}_{max}$ are the maximum value of $RMSE$, $MAE$, and $MAEP$ computed by seven optimizers.

In this study an effective graphical method called Taylor diagram^49^ is used to better show the efficiency of proposed method compared with the other methods. To implement this graph, it is used three performance indices, including standard deviation (SD), correlation coefficient (R), and centered root mean square error ($cRMSE$). This graph is plotted in a polar space in which its geometrical distance to the target point indicates the efficiency of each method^49^. The $cRMSE$ can be formulated as,

37 $${cRMSE}^{2}={SD}_{LP}^{2}+{SD}_{Alg}^{2}-2\times R\times {SD}_{LP}\times {SD}_{Alg},$$

where ${SD}_{LP}$ and ${SD}_{Alg}$ refers the standard deviation of the optimal values of releases caluclated by the LP and optimization algorithms, respectively.

## Results and discussion

### Results of ten-reservoir problem

Optimal results of ten-reservoir problem calculated by all certain optimization techniques are pointed out in this section. The problem was solved by using LINGO 8.0 software and the global solution was 1194.44^30,50^. The control parameter amounts of all optimizers for the problem are illustrated in Table 1. Also, the population size and $MaxNFE$ for this problem are equal to100 and 600,000, respectively. Table 2 shows the outcomes achieved by the seven competitors, including the best, worst, mean, and SD for this problem in 30 different experiments. For ten-reservoir problem, only SATLDE can achieve the best values on four metrics (i.e., the best (1196.98), worst (1194.00), mean (1195.85), and SD (0.84)). A polar space is used to produce this graph, and the geometrical distance between the target point and the graph’s origin represents the effectiveness of each approach. From the results, it can powerfully certificate the importance of the proposed SATLDE in optimizing complicated multi-reservoir optimization problem. It should be noted that for this problem the value of global optimum is 1194.44, while the best value obtained by the SATLDE is 1196.98. Therefore, there are a difference between the optimum value achieved by the LP and SATLDE. This difference is due to the PF coefficients considered for this problem^30,50,51^. In fact, the PF in Eq. (23) is not equal to zero, because the PF coefficient is small for this problem. Similar to the previous problem, the value of PF coefficient is not changed in this study, because this problem is a benchmark problem.

**Table 1:** Control parameters of all optimizers.

Algorithm	Parameters
SATLDE	$Np=100$
jDE	$Np=100$
HSLSO	$Np=100$, *Crossover rate* = 0.5
ITLBO	$Np=100$
MLBSA	$Np=100$
MS-DEPSO	$Np=100$, ${Cr}_{min}=0.3$, ${Cr}_{max}=0.5$
SATLBO	$Np=100$

**Table 2:** Results of SATLDE algorithm and six other optimizers on four-reservoir system.

	Best	Worst	Mean	SD
**SATLDE**	**1196.98**	**1194.00**	**1195.85**	**0.84**
MLBSA	1168.20	1121.48	1144.87	12.34
HSLSO	1142.39	1039.04	1101.23	29.83
ITLBO	1091.07	1014.41	1057.05	17.17
jDE	1133.47	1094.44	1111.66	9.51
MS-DEPSO	1187.02	1166.35	1180.79	4.66
SATLBO	1138.69	1103.51	1121.16	9.42

Significant values are in bold.

Table 3 presents the performance of seven optimization algorithms in this study, which are comprehensively evaluated by six performance metrics (i.e., R, RMSE, MAE, MAPE, E, and IA). The obtained results reported in Table 5 indicated that the SATLDE algorithm has the highest correlation and lowest error (R = 0.995, RMSE = 0.276, MAE = 0.061, MAPE = 4.809, E = 0.989, IA = 0.997) compared with the optimal value calculated by the other optimization algorithms.

**Table 3:** Performance metrics of SATLDE algorithm and six other optimizers for ten-reservoir problem.

	SATLDE	HSLSLO	ITLBO	jDE	MS-DEPSO	SATLBO	MLBSA
R	0.995	0.914	0.805	0.729	0.818	0.944	0.866
RMSE	0.276	1.088	1.589	1.848	1.554	0.893	1.348
MAE	0.061	0.518	1.076	1.291	0.868	0.337	0.967
MAPE	4.809	2660.826	6605.713	10,438.024	3599.993	1846.592	7427.983
E	0.989	0.835	0.647	0.523	0.663	0.889	0.746
IA	0.997	0.955	0.889	0.850	0.903	0.971	0.919

**Figure 6:**
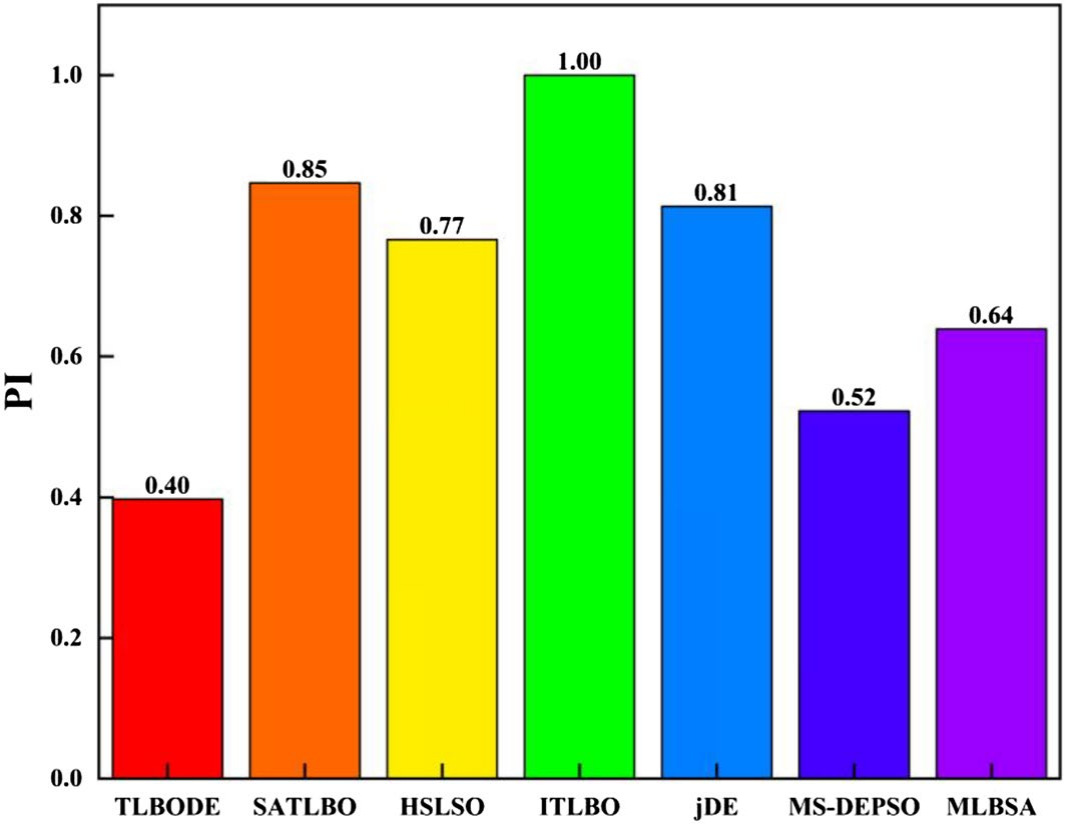
Comparing PI metric for SATLDE and other optimizers for four-reservoir problem.

**Figure 7:**
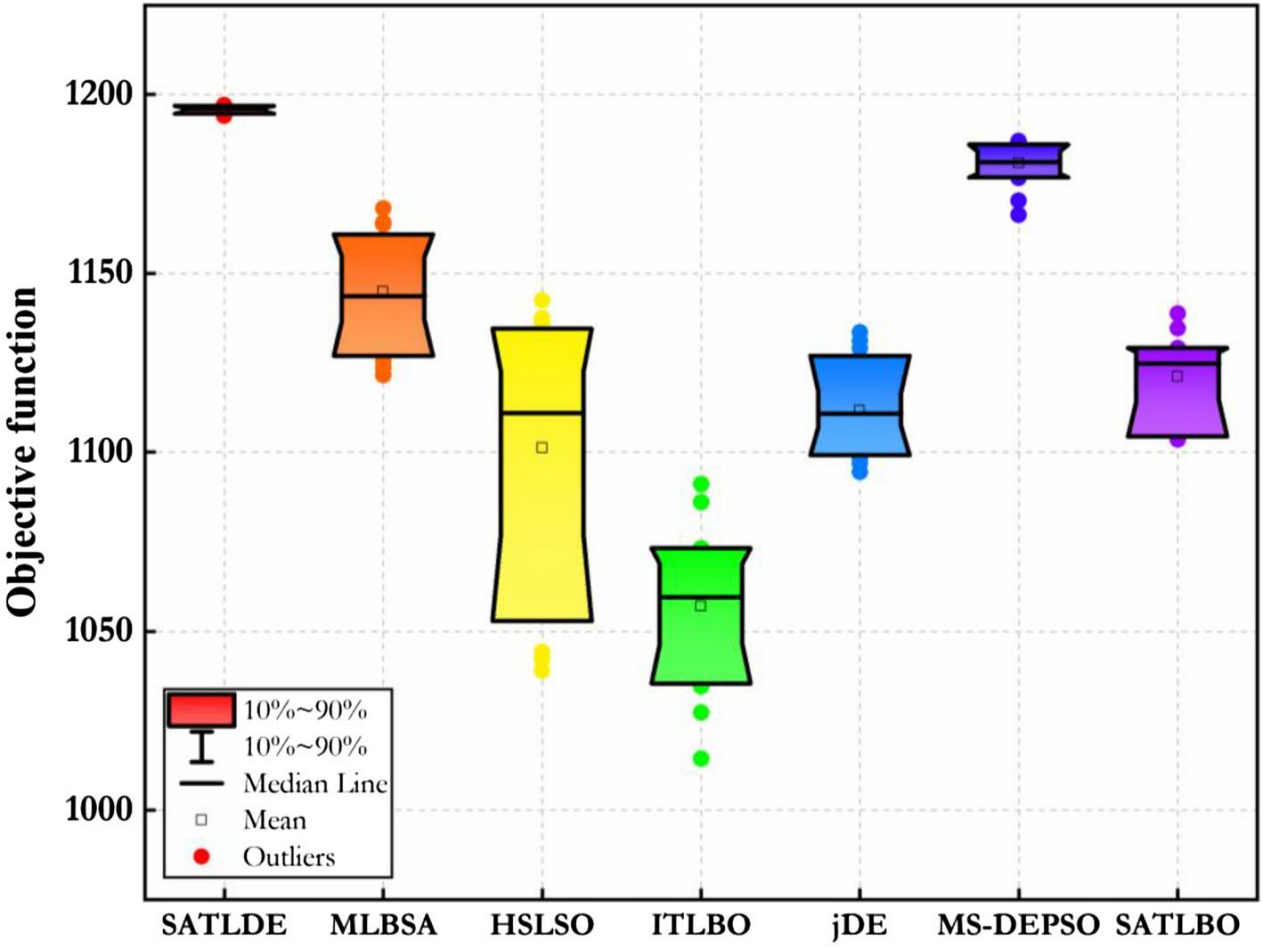
Boxplots of all optimizers for ten-reservoir problem.

According to Fig. 6, SATLDE has a superior performance compared to its competitors MS-DEPSO (0.52), MLBSA (0.64), HSLSO (0.77), jDE (0.81), SATLBO (0.85), and ITLBO (1.00). Consequently, it can be concluded based on this index that, the optimal results calculated by SATLDE have a significant compliance with the global optimal solution computed by the LP. The boxplot of all optimizers in Fig. 7 shows that SATLDE illustrates a superior performance compared with other optimizers with regard to the solution distribution.

**Figure 8:**
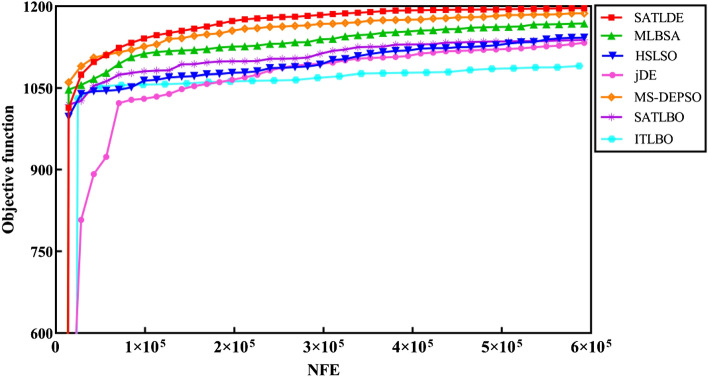
Convergence curves of all optimization techniques for ten-reservoir problem.

**Figure 9:**
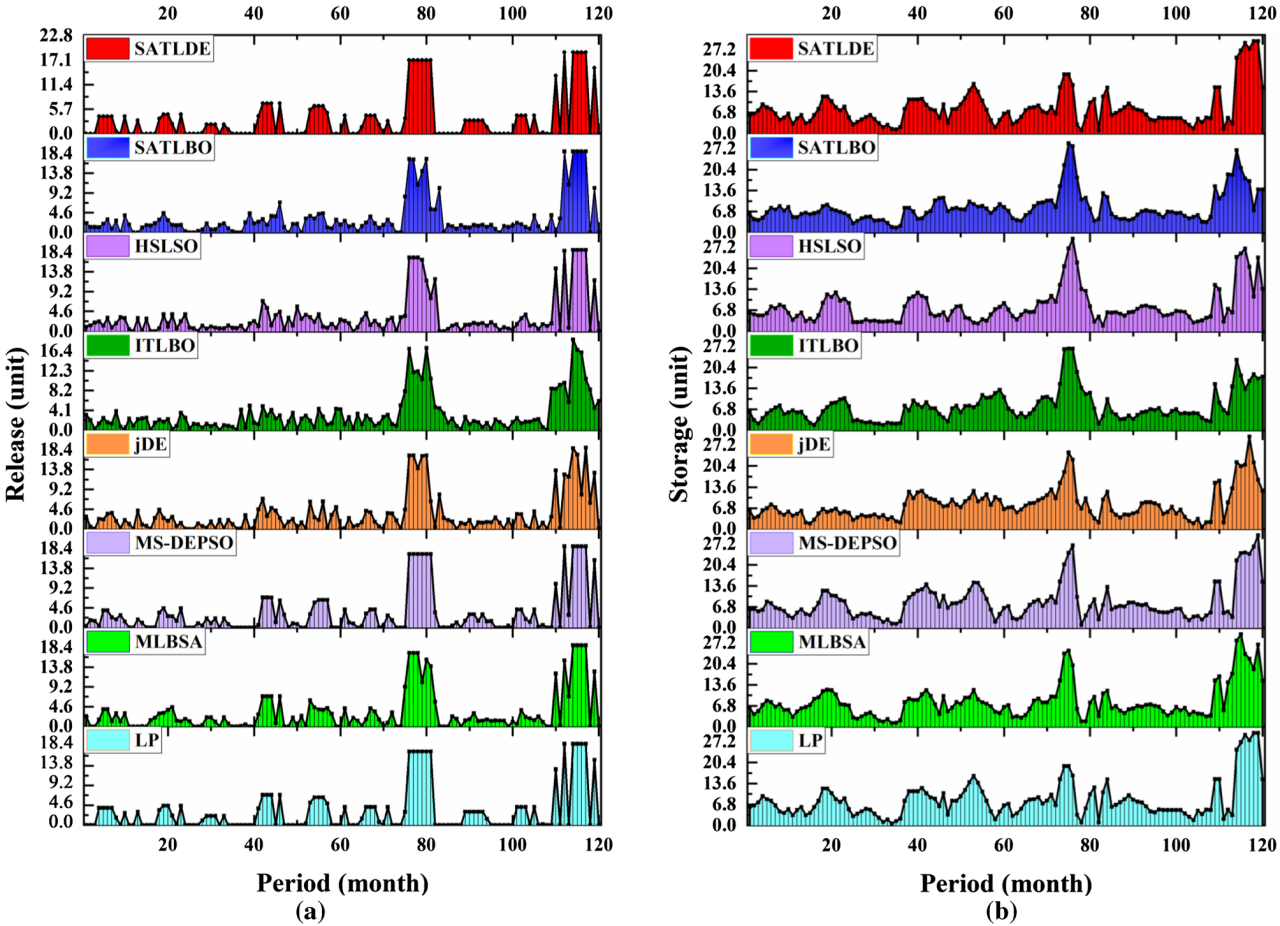
Reservoir volumes for ten-reservoir problem at two modes: (**a**) storage, (**b**) release.

The convergence graphs in Fig. 8 show that SATLDE has the fastest convergence speed compared with the other competitors for the ten-reservoir problem. Accordingly, it can be inferred SATLDE can achieve precise and reliable reservoir releases compared to the others. Figure 9 shows the monthly volume of reservoir releases and storage optimized by SATLDE and top two other optimization algorithms (i.e., SATLBO, MS-DEPSO, and MLBSA). Visually, the optimal release and storage achieved by SATLD have the significant correspondence with the global optimal values calculated by LP.

**Figure 10:**
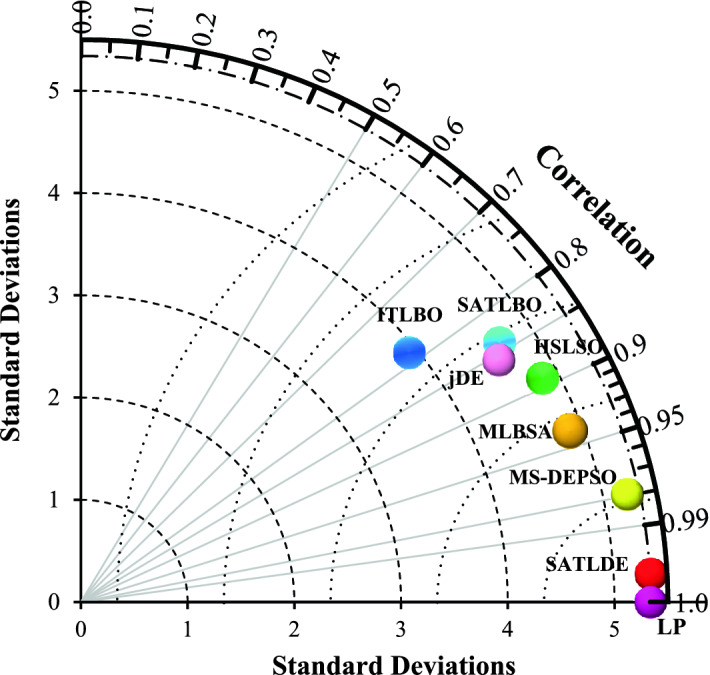
Taylor diagram of all optimizers for ten-reservoir problem.

The Taylor diagram of all optimization algorithms is depicted in Fig. 10, showing that, the proposed algorithm is closer to the global optimal solution (target point) compared to the other algorithms. This graph highlights once again the capacity of SATLDE to handle complex multi-reservoir systems.

### Ranking analysis

In this section, two non-parametric statistical tests namely the Friedman^52^ and Wilcoxon Signed Rank (WSR)^52^ tests are used to show the best method for optimizing multi-reservoir systems. Table 4 gives the results of Friedman test for two multi-reservoir problems. According to this test, SATLD has the best rank (1) compared with the six other optimizers.

**Table 4:** Results of Friedman test for the ten-reservoir problems.

Algorithm	Friedman
**SATLDE**	**1.00**
HSLSLO	5.50
ITLBO	7.00
jDE	4.50
MS-DEPSO	2.00
SATLBO	5.00
MLBSA	3.00

Significant values are in bold.

Based on the WSR, *R*^+^ illustrates the sum of ranks over all different runs in which SATLDE outperformed the contestant optimizer, while *R*^−^ represents the sum of ranks over all runs where the contestant optimizer outperformed the SATLDE. In addition, in a statistical hypothesis test ($\alpha$ = 0.05), the p-value detects the importance of the outcomes. The results showed that the values of *R*^+^ and *R*^−^ obtained by WSR for the proposed method compared to other optimization methods are the same and equal 465 and 0, respectively. Accordingly, it can be said that SATLDE performs significantly better than the other optimizers.

## Results of real-world multi-reservoir system

Results of the multi-reservoir system as a real-world case study are provided in this section in detail. As stated in “Real-world multi-reservoir system” section, this system consists of four reservoirs and its main purpose is hydropower generation.

### Statistical criteria comparison

To indicate the robustness of the SATLDE, the statistical results of seven optimizers (i.e., HSLSO, MS-DEPSO, jDE, MLBSA, ITLBO, SATLBO) are reported in Table 5, comprising the best, mean, worst, and SD of the fitness function values. Seven optimization algorithms are independently run 30 times, while the $Np$ and $MaxNFE$ are set to 100 and 1000, respectively. The problem was optimized over a 55-year operational time step.

**Table 5:** Results of SATLDE algorithm and six other optimizers for real world multi-reservoir system.

	Best	Worst	Mean	SD
**SATLDE**	207.63	255.08	223.63	9.88
HSLSLO	238.59	307.14	263.08	19.53
ITLBO	228.89	298.27	260.84	14.53
jDE	470.09	731.55	582.97	59.74
MS-DEPSO	290.75	342.63	319.89	13.96
SATLBO	274.83	329.78	296.17	14.44
MLBSA	278.73	402.52	326.60	28.34

Significant values are in bold.

As demonstrated in Table 5, it can be obviously found that the best (207.63), mean (223.63), and worst (255.08) of the fitness function values obtained by SATLDE are obviously better than the control optimizers. In addition, SATLDE makes about 49%, 32%, 83%, 29%, 32%, and 65% improvements in the SD values compared with HSLSO, ITLBO, jDE, MS-DEPSO, SATLBO, and MLBSA, respectively. Besides, from Table 6 also finds that the SATLDE is more reliable and accurate than other optimizers according to the performance.

**Table 6:** Generated power by seven optimizers for multi-reservoir system.

Method	Karoun3	Karoun1	Godar	Dez	Total
**SATLDE**	**6.35E+05**	**8.46E+05**	**8.51E+05**	**2.78E+05**	**2.61E+06**
MLBSA	6.34E+05	7.86E+05	7.85E+05	2.60E+05	2.46E+06
HSLSO	6.35E+05	8.22E+05	8.19E+05	2.76E+05	2.55E+06
ITLBO	5.12E+05	7.50E+05	6.22E+05	2.27E+05	2.11E+06
jDE	5.90E+05	7.70E+05	7.68E+05	2.60E+05	2.39E+06
MS-DEPSO	6.26E+05	8.03E+05	8.30E+05	2.68E+05	2.53E+06
SATLBO	6.31E+05	7.83E+05	8.12E+05	2.46E+05	2.47E+06

Significant values are in bold.

### Box plot analysis

**Figure 11:**
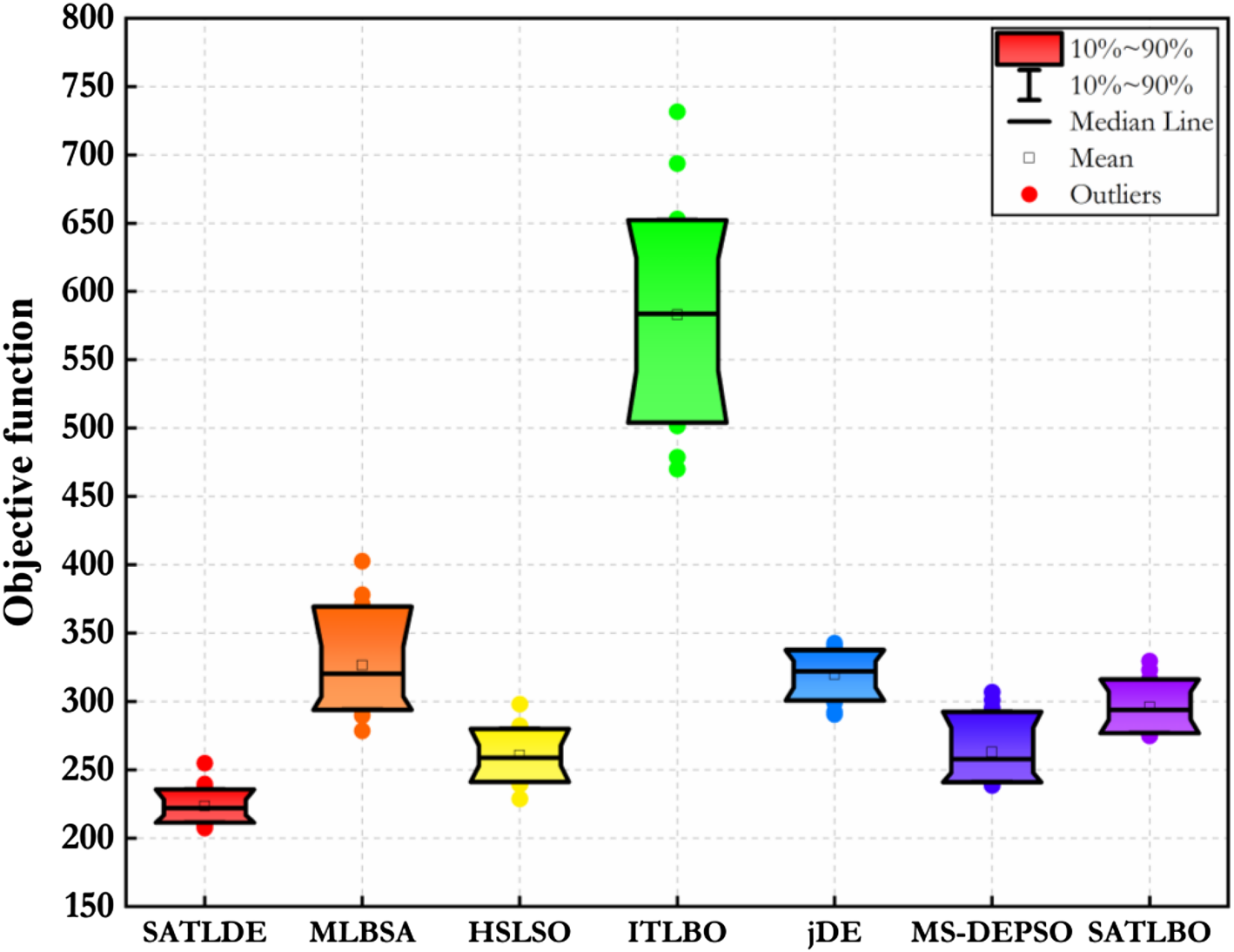
Boxplots of all optimizers for real world multi-reservoir problem.

Box plot graph is used to illustrate the variation rate of the fitness function calculated by the proposed SATLDE and other optimization methods during 30 different runs. Figure 11 depicts the Box plot of 7 optimizers for the multi-reservoir system. The figure indicates the seven optimization algorithms’ maximum, minimum, and average fitness functions. In addition, the Box plot shows the density range of fitness function over 30 runs. It can be seen that compared with six other optimization algorithms, the location of SATLDE is clearly lower, and the range of distribution is smaller, indicating the priority of the optimization technique. Therefore, SATLDE can be utilized as an efficient tool for optimizing multi-reservoir problems.

### Convergence trajectory analysis

**Figure 12:**
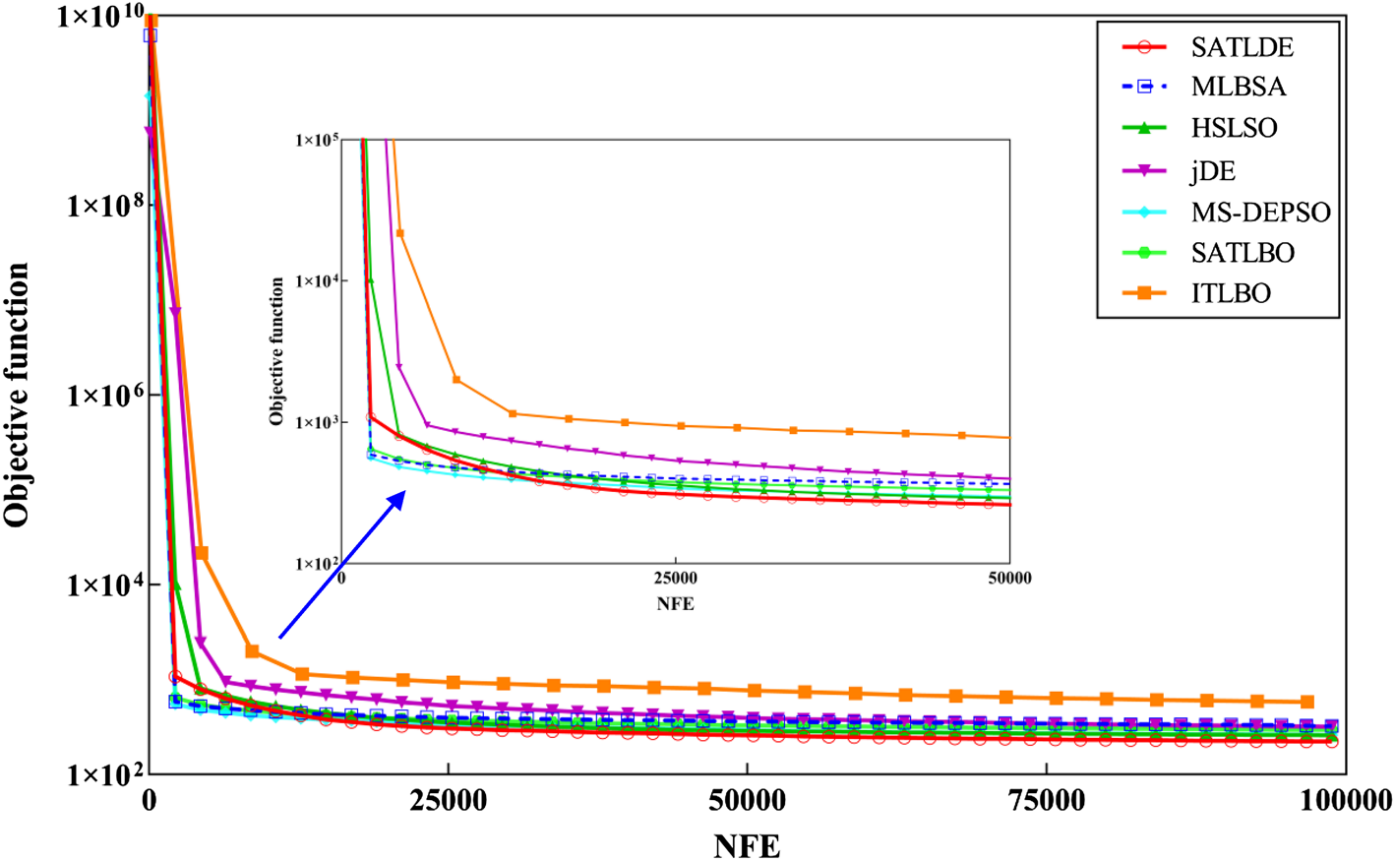
Convergence graphs of all optimizers for the multi-reservoir system.

Figure 12 illustrates the convergence graphs of seven methods for the multi-reservoir problem. The figure displays the average values of the fitness function for all optimizers, obtained in 30 different runs. It has been discovered that the SATLDE has a faster convergence speed than the other approaches after 12,500 NFE. In fact, the proposed method can quickly explore feasible solutions by increasing the NFE. The high accuracy and speed up of the SATLDE convergence are due to the adaptive parameters and different strategies added to the proposed algorithm.

### Detailed hydropower production analysis

Table 6 gives the total power generated by all optimization methods for each reservoir of the multi-reservoir system. It can be found from the table that the power generated by SATLDE for Karoun3 (6.35E+05 MW), Karoun1 (8.46E+05 MW), Godar (8.51E+05 MW), and Dez (2.78E+05 MW) are more than those produced by the other methods. In addition, the total power generated by SATLDE (2.61E+06 MW) is more than the MLBSA (2.46E+06), HSLSO (2.55E+06), ITLBO (2.11E+06 MW), jDE (2.39E+06 MW), MS-DEPSO (2.53E+06 MW), and SATLBO (2.47 + 06 MW), respectively. Based on the results, the best optimization method to produce power is the SATLDE, followed by the HSLSO, MS-DEPSO, SATLBO, MLBSA, jDE, and ITLBO, respectively.

**Figure 13:**
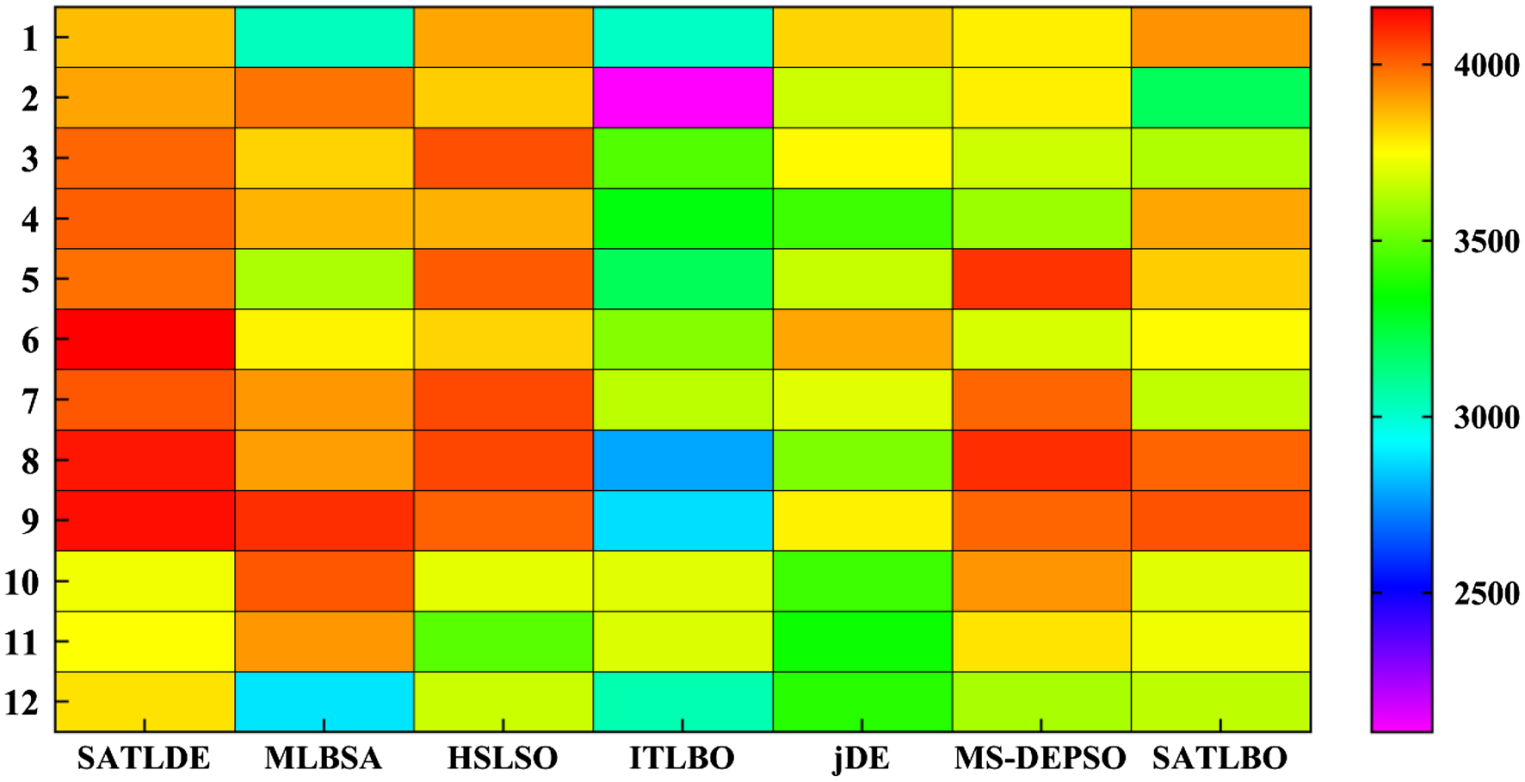
Monthly average of hydropower energy generated by all optimizers.

Figure 13 displays the monthly distribution of power generated by all optimizers in the form of a heat map plot. It can be observed that the proposed SATLDE can produce more monthly power than those generated by the six other optimizers. It is worth noting that in the SATLDE, most of the power generated occurs in months 1 to 9 (January to September).

**Figure 14:**
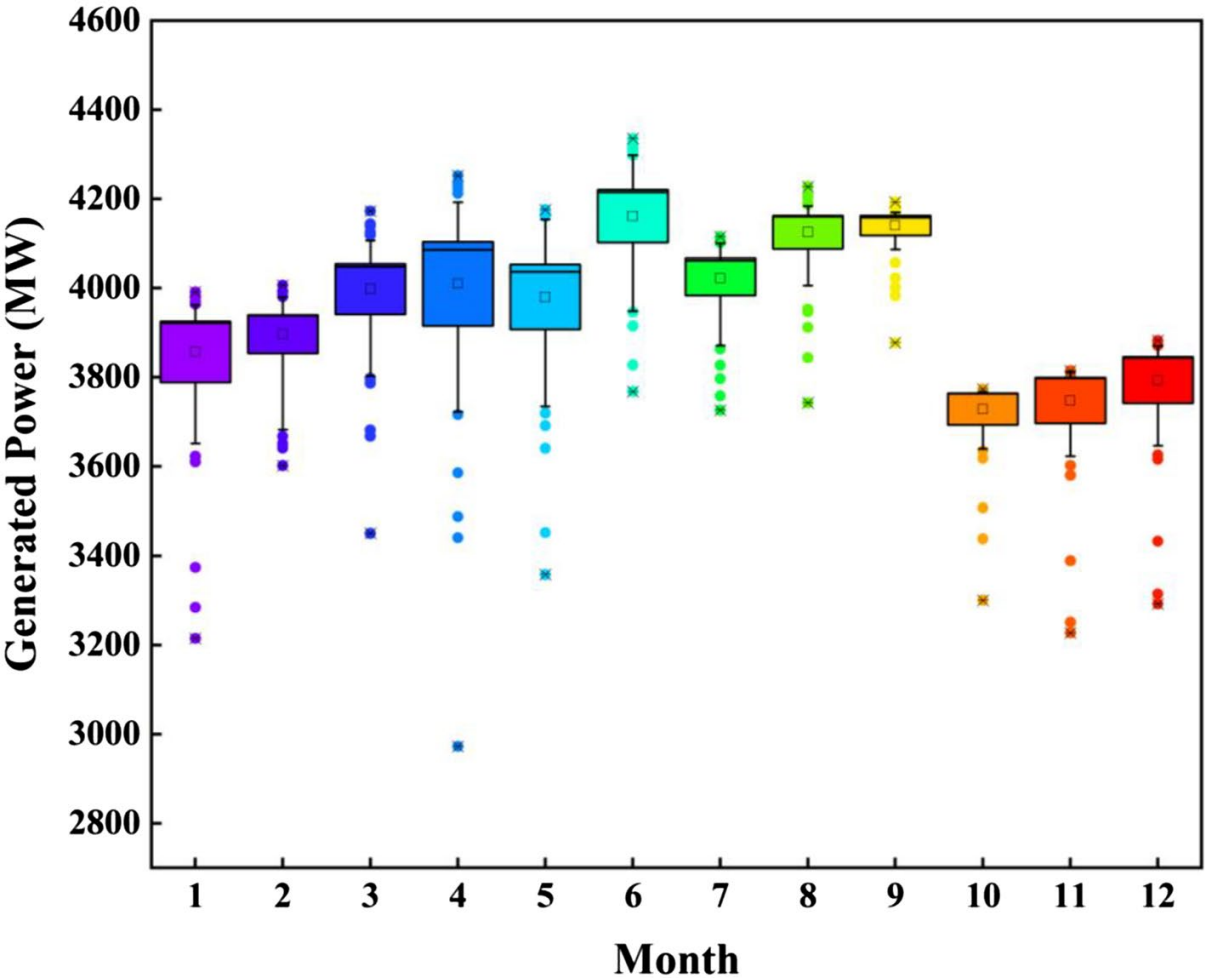
Monthly generated power gained by SATLDE.

**Figure 15:**
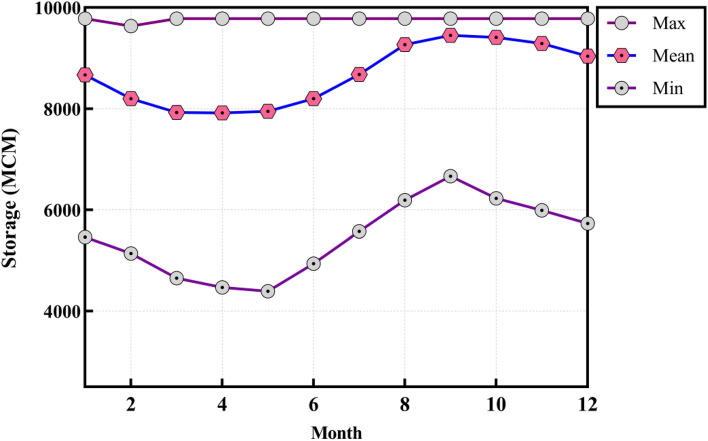
Monthly maximum, minimum, and average of reservoir storage gained by SATLDE.

Figure 14 depicts the distribution of monthly power generated by SATLDE in the form of a Box plot. Based on the figure, the maximum and minimum energy produced belong to the 6th and 10th, respectively. Moreover, the monthly maximum, minimum, and average volumes of reservoir storage achieved by the proposed method are shown in Fig. 15. According to the findings of this study, SATLDE outperforms the MS-DEPSO, HSLSO, MLBSA, jDE, and ITLBO in determining the optimal power generated from the multi-reservoir system. This is the case, although all six optimizers can be used effectively to reach optimal operating rules for the multi-reservoir system successfully.

## Conclusion

A self-adaptive teaching learning-based with differential evolution (SATLDE) is developed for optimizing hydropower multi-reservoir systems. SATLDE uses a ranking probability mechanism to select the teacher or learner stage; then a boosted teacher stage utilizing various teaching ways is applied to move the learners to enhance themselves based on their levels. Finally, an efficient mutation operator is employed to the learner stage for improving global search capability. In addition, an adaptive method is used for parameter settings. SATLDE applies a learner factor according to the adaptive approach to store the optimal control parameter values at each iteration.

The efficiency of SATLDE has been evaluated by optimizing a well-known benchmark multi-reservoir system, i.e., the ten-reservoir problem. For the ten-reservoir problem, SATLDE shows superior results, with the best fitness function value equal to 1196.98, while MLBSA, HSLSO, ITLBO, jDE, MS-DEPSO, and SATLBO can converge to 1168.20, 1142.39, 1091.07, 1133.47, 1187.02, and 1138.69, respectively. SATLDE demonstrates significant efficiency in reliability, precision, and robustness. Therefore, the proposed algorithm can be employed in other multi-reservoir systems in order to quickly extract the optimal operating rules for multi-reservoir systems, thus improving energy efficiency.

To exhibit the ability of SATLDE in optimizing real-world problems, a multi-reservoir system consists of four reservoirs located in Iran was used as a case study. In this research, a nonlinear operating rule is developed to maximize the energy produced from the multi-reservoir system. In fact, SATLDE and six other optimizers are applied to derive the optimal operating rules. It statistical analysis of results shows that, SATLDE can present the superior ability in obtaining more promising solutions as compared with the six optimization methods. In addition, SATLDE could produce remarkably more power (2610 GW) than the MLBSA (2460 GW), HSLSO (2550 GW), ITLBO (2110 GW), jDE (2390 GW), MS-DEPSO (2530 GW), and SATLBO (2470 GW). Consequently, results show that SATLDE is able to present more reliable and accurate operating rules for the multi-reservoir system. Accordingly, it can be considered an effectual alternative for optimizing operating rules of other complex hydropower multi-reservoir systems.

For future study, the proposed SATLDE can be ensembled with other optimization methods such as the GBO algorithm. Moreover, we will investigate the ability of proposed method to derive optimal parameters of photovoltaic models. In addition, although the data in the present study shows both wet and dry periods, we recommend that future work focus on including climate change scenarios to ensure that the operating rules can respond to a broader range of climate conditions. Finally, SATLDE can be used for solving machine learning problems.

## Data Availability

All data used in the study are available from the corresponding author by request.

## Code availability

The source codes of the paper and online web service for any question and Supplementary Material including all equations of the algorithms will be publicly available at http://imanahmadianfar.com.
